# Role of Nuclear Claudin-4 in Renal Cell Carcinoma

**DOI:** 10.3390/ijms21218340

**Published:** 2020-11-06

**Authors:** Takuya Owari, Takamitsu Sasaki, Kiyomu Fujii, Rina Fujiwara-Tani, Shingo Kishi, Shiori Mori, Takuya Mori, Kei Goto, Isao Kawahara, Yasushi Nakai, Makito Miyake, Yi Luo, Nobumichi Tanaka, Masuo Kondoh, Kiyohide Fujimoto, Hiroki Kuniyasu

**Affiliations:** 1 Department of Molecular Pathology, Nara Medical University, 840 Shijo-cho, Kashihara, Nara 634-8521, Japan; tintherye@gmail.com (T.O.); takamitu@fc4.so-net.ne.jp (T.S.); toto1999-dreamtheater2006-sms@nifty.com (K.F.); rina_fuji@naramed-u.ac.jp (R.F.-T.); nmu6429@yahoo.co.jp (S.K.); shi.m.0310@i.softbank.jp (S.M.); pt_mori_t@yahoo.co.jp (T.M.); ilgfgtk@gmail.com (K.G.); isao_kawahara@a011.broada.jp (I.K.); lynantong@hotmail.com (Y.L.); 2 Department of Urology, Nara Medical University, 840 Shijo-cho, Kashihara, Nara 634-8522, Japan; nakaiyasusiuro@live.jp (Y.N.); makitomiyake@yahoo.co.jp (M.M.); sendo@naramed-u.ac.jp (N.T.); 3 Key Laboratory of Neuroregeneration of Jiangsu and Ministry of Education, Co-Innovation Center of Neuroregeneration, Nantong University, Nantong 226001, Jiangsu, China; 4 Drug Innovation Center, Graduate School of Pharmaceutical Sciences, Osaka University, 6-1 Yamadaoka, Suita, Osaka 565-0871, Japan; claudindds@gmail.com

**Keywords:** CLDN4, PKCε, EphA2, Ephrin A1, EMT

## Abstract

Claudin-4 (CLDN4) is a tight junction protein to maintain the cancer microenvironment. We recently reported the role of the CLDN4 not forming tight junction in the induction of epithelial-mesenchymal transition (EMT). Herein, we investigated the role of CLDN4 in renal cell carcinoma (RCC), focusing on CLDN4. CLDN4 expression in 202 RCCs was examined by immunostaining. CLDN4 phosphorylation and subcellular localization were examined using high metastatic human RCC SN12L1 and low metastatic SN12C cell lines. In 202 RCC cases, the CLDN4 expression decreased in the cell membrane and had no correlation with clinicopathological factors. However, CLDN4 was localized in the nucleus in 5 cases (2%), all of which were pT3. Contrastingly, only 6 of 198 nuclear CLDN4-negative cases were pT3. CLDN4 was found in the nuclear fraction of a highly metastatic human RCC cell line, SN12L1, but not in the low metastatic SN12C cells. In SN12L1 cells, phosphorylation of tyrosine and serine residues was observed in cytoplasmic CLDN4, but not in membranous CLDN4. In contrast, phosphorylation of serine residues was observed in nuclear CLDN4. In SN12L1 cells, CLDN4 tyrosine phosphorylation by EphA2/Ephrin A1 resulted in the release of CLDN4 from tight junction and cytoplasmic translocation. Furthermore, protein kinase C (PKC)-ε phosphorylated the CLDN4 serine residue, resulting in nuclear import. Contrarily, in SN12C cells that showed decreased expression of EphA2/Ephrin A1 and PKCε, the activation of EphA2/EphrinA1 and PKCε induced cytoplasmic and nuclear translocation of CLDN4, respectively. Furthermore, the nuclear translocation of CLDN4 promoted the nuclear translocation of Yes-associated protein (YAP) bound to CLDN4, which induced the EMT phenotype. These findings suggest that the release of CLDN4 by impaired tight junction might be a mechanism underlying the malignant properties of RCC. These findings suggest that the release of CLDN4 by impaired tight junction might be one of the mechanisms of malignant properties of RCC.

## 1. Introduction

The frequency of renal cell carcinoma (RCC) in the Japanese population is 6 out of 100,000, accounting for approximately 1% of all cancers [1] and 2% of all cancers in North America [2]. Thus, RCC is not a frequent disease; however, up to 30% of RCC cases have synchronous or metachronous metastases [2]. Furthermore, RCC cells were detected in the bone marrow aspiration in 25% of RCC cases without metastasis [3], suggesting that RCCs have a high metastatic potential. In cases with clear cell RCC (CCC), which is the most common RCC, 90% or more showed chromosome 3P deletion and inactivation of the von Hippel-Lindau (VHL) gene, leading to hypoxia-inducible factor (HIF)-1 activation [4]. Epithelial-mesenchymal transition (EMT) is a factor related to the metastatic potential of CCC [5], and HIF1 activation is considered a factor promoting EMT [6]. Claudin-4 (CLDN4) is a tight junction protein and an epithelial marker [7,8], and its decreased expression correlates with EMT [9].

Attention has recently been paid to the function of the non-tight junction protein claudins (CLDNs). CLDNs are normally localized in the cell membrane as a tight junction-forming protein, and they regulate the diffusion of solutes through the intercellular space by their homotypic binding [7]. In contrast, it has been reported that CLDNs that do not form a tight junction take part in intracellular signaling [10]. CLDN7 binds to integrin β1 and suppresses the growth and movement of colon cancer cell lines [11,12]. CLDN4, which does not form tight junctions in undifferentiated gastric cancer, becomes a ligand for integrin β1, activates focal adhesion kinase, and promotes stemness [13]. Furthermore, we have reported that impairment of tight junctions caused by *Clostridium perfringens* enterotoxin (CPE) leads to alterations in CLDN4 localization, activates Yes-associated protein (YAP), and induces EMT [14,15,16]. However, significance of nuclear CLDN4 has not been reported in RCC. 

In this study, we investigated the role of CLDN4 in RCC, focusing on nuclear CLDN4.

## 2. Results

### 2.1. Expression of CLDN4 in Renal Cell Carcinomas

**Figure 1:**
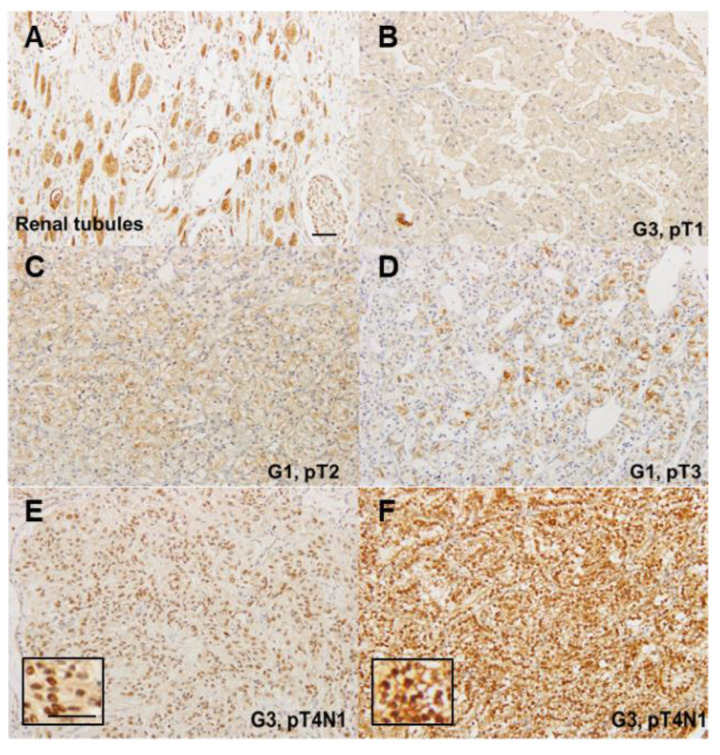
Immunohistochemistry of CLDN4 in renal cell carcinomas. (**A**) Non-tumoral renal tissue. Immunoreactivity of CLDN4 is observed in the cytoplasmic membrane and cytoplasm. (**B**–**F**) Expression of CLDN4 in renal cell carcinomas. (**B**–**D**) Faint membranous and scattered cytoplasmic staining of CLDN4. (**E**,**F**) Diffuse nuclear staining of CLDN4. Insert, high magnification image. Scale bar, 100 μm. G, histological grade; pT, local progression of primary tumor; pN, lymph node metastasis.

First, CLDN4 expression was examined by immunostaining (Figure 1). As shown in Figure 1A, CLDN4 expression was observed in the cell membrane and cytoplasm of normal tubular epithelium. In RCCs, expression of CLDN4 was found weakly in the cell membrane and scattered in the cytoplasm (Figure 1B–D). The relationship between CLDN4 expression on the cell membrane and clinicopathologic features was then examined (Table 1). CLDN4 expression was not correlated with histological type, histological grade, cancer progression (pT), lymph node metastasis (pN), or stage.

CLDN4 expression was diffusely observed in the nuclei of cancer cells in 5 out of 202 cases (Figure 1E,F), all of which were pT3 (Table 2). In contrast, only 3% (6 out of 198 cases) had pT3 in the cases where CLDN4 was localized in the cell membrane and cytoplasm. For cases wherein CLDN4 expression was observed in the nucleus, the cancer was significantly advanced.

### 2.2. Role of EphA2 in CLDN4 Cytoplasmic Translocation

**Figure 2:**
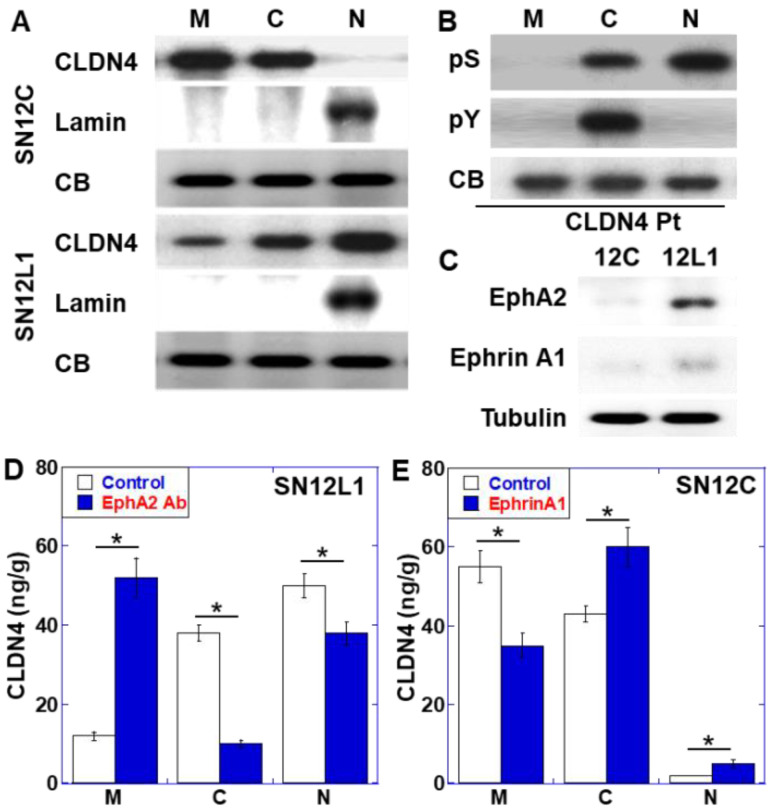
Effect of EphA2/Ephrin A1 on the subcellular localization of CLDN4 in renal cell carcinoma cell line. (**A**) Subcellular localization of CLDN4 in SN12C (low metastatic parental cell line) and SN12L1 (high metastatic subline of SN12C cells). Lamin was added as a nuclear marker. (**B**) Phosphorylation property of CLDN4 in SN12L1 cells. Immunoprecipitant was detected using anti-pS or anti-pY antibody. (**C**) Expression of EphA2 and Ephrin A1 examined using immunoblotting. (**D**) Alteration of the subcellular localization of CLDN4 observed using anti-EphA2 antibody (1 μg/mL for 24 h) in SN12L1 cells. CLDN4 protein level was measured using enzyme-linked immunosorbent assay (ELISA). (**E**) Alteration of the subcellular localization of CLDN4 using Ephrin A1 (10 μg/mL for 24 h) in SN12C cells. CLDN4 protein level was measured using ELISA. Error bar, standard deviations calculated by ordinary analysis of variance from three independent examinations. M, membrane fraction; C, cytosol fraction; N, nuclear fraction; CB, Coomassie blue; pS, phosphoserine; pY, phosphotyrosine; Pt, immunoprecipitated; PKC-I, PKCε inhibitor peptide; PKC-A, PKCε activator; WL, whole-cell lysate; Nuclear, nuclear fraction.

To elucidate the significance of CLDN4 in the nucleus, we compared in the intracellular localization of CLDN4 between the highly metastatic SN12L1 cell line with the parent low metastatic SN12C cell line (Figure 2). CLDN4 was localized in the cell membrane and cytoplasm in both cell lines, whereas it was localized in the nucleus only in SN12L1 cells (Figure 2A). The phosphorylation of serine or tyrosine residues in CLDN4 immunoprecipitates in the membrane, cytoplasmic, and nuclear fractions of SN12L1 cells was examined; however, no phosphorylation was observed in the membrane fraction (Figure 2B). In contrast, the phosphorylation of both serine and tyrosine was observed in the cytoplasmic fraction, while the nuclear fraction showed only serine phosphorylation.

EphA2 is reported to phosphorylate CLDN4 at Tyr208 [18]. We examined the expression of EphA2 and the ligand Ephrin A1 (Figure 2C). SN12C cells expressed EphA2 and Ephrin A1 at very low levels, whereas SN12L1 cells expressed both genes at high levels. In SN12L1 cells, the inhibition of EphA2 altered CLDN4 intracellular localization and resulted in an increase of CLDN4 in the membrane fraction and decrease in the cytosolic and nuclear fractions (Figure 2D). In contrast, in SN12C cells, treatment with Ephrin A1 altered CLDN4 intracellular localization, such that there was a decrease in the membrane fraction and an increase in the cytosolic and nuclear fractions (Figure 2E).

### 2.3. Role of PKCε in CLDN4 Nuclear Translocation and YAP Activation

**Figure 3:**
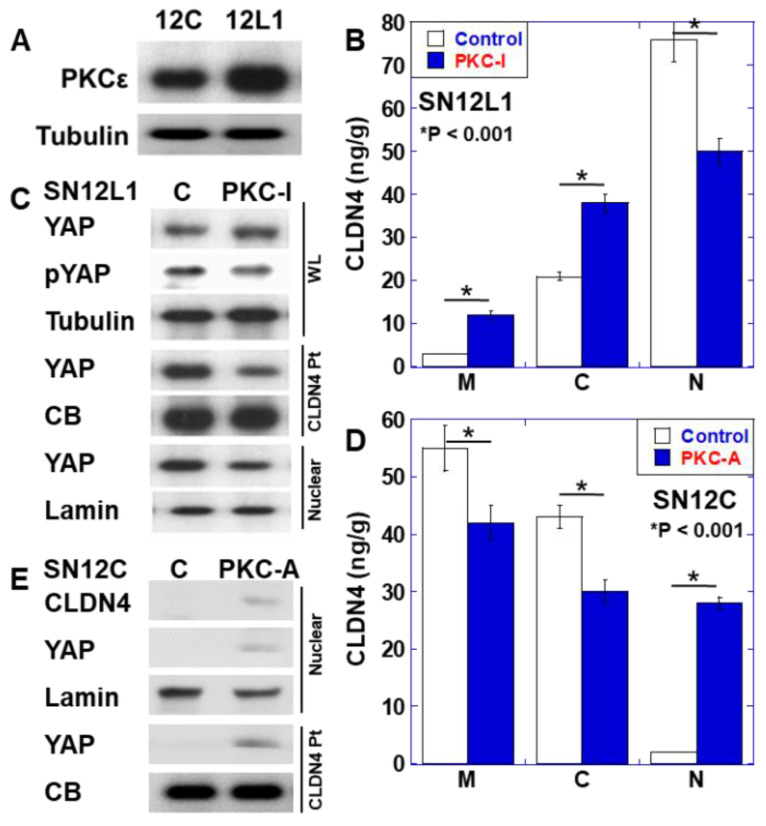
Effect of PKCε on the nuclear localization of CLDN4 and Yes-associated protein (YAP) activation in renal cell carcinoma cell line. (**A**) Expression of PKCε examined using immunoblotting. (**B**) Alteration of the subcellular localization of CLDN4 by PKC-I (10 μM for 24 h) in SN12L1 cells. CLDN4 protein level was measured using enzyme-linked immunosorbent assay (ELISA). (**C**) YAP inactivation in SN12L1 cells treated with PKC-I. (**D**) Alteration of the subcellular localization of CLDN4 using PKC-A (5 μM for 24 h) in SN12C cells. CLDN4 protein level was measured using ELISA. (**E**) YAP activation in SN12C cells treated with PKC-A. Error bar, standard deviations calculated by ordinary analysis of variance from three independent examinations. M, membrane fraction; C, cytosol fraction; N, nuclear fraction; CB, Coomassie blue; Pt, immunoprecipitated; PKC-I, PKCε inhibitor peptide; PKC-A, PKCε activator; WL, whole-cell lysate; Nuclear, nuclear fraction; pYAP, phosphorylated YAP.

As PKCε has been reported to phosphorylate the serine residue of CLDN4 [19], the expression of PKCε was examined (Figure 3A). Expression of PKCε was observed in SN12L1 cells, but not in SN12C cells. Furthermore, treatment of SN12L1 cells with a PKCε inhibitor increased CLDN4 expression in the plasma membrane and cytoplasmic fractions, whereas its levels in the nuclear fraction decreased to 40% (Figure 3B). We previously reported that nuclear CLDN4 is correlated with the activation of YAP in oral cancer [14]. Therefore, we investigated YAP in SN12L1 cells (Figure 3C). YAP was bound to CLDN4 in whole-cell lysate, and nuclear translocation of YAP was observed. Notably, treatment with PKCε did not change the YAP protein level, but it decreased CLDN4-bound YAP and nuclear YAP. In contrast, when PKCε activator was applied to SN12C cells, in which nuclear CLDN4 was not observed, the intracellular localization of CLDN4 decreased in the plasma membrane and cytoplasmic fraction but increased in the nuclear fraction (Figure 3D). Moreover, under these conditions, we observed binding between YAP and CLDN4, and nuclear YAP was induced (Figure 3E).

### 2.4. YAP Activation and Induction of the EMT Phenotype

**Figure 4:**
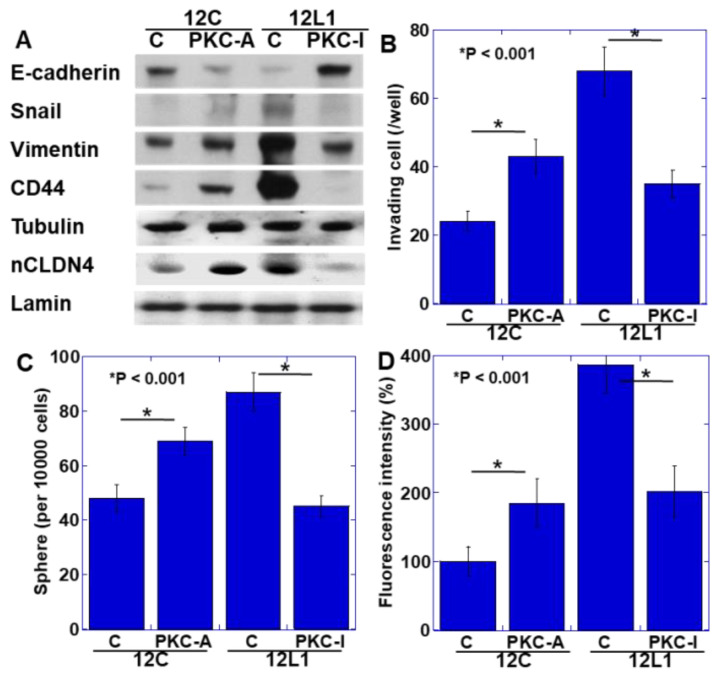
Effect of PKCε activity on malignant phenotypes in human renal cell carcinoma cell line. (**A**) Effect of PKC-A or PKC-I on the epithelial-mesenchymal transition (EMT) phenotype examined by immunoblotting of EMT-related proteins. (**B**–**D**) Effect of PKC-A or PKC-I on invasion activity (**B**), sphere-forming activity (**C**), and lung metastasis (**D**). Error bar, standard deviations calculated by ordinary analysis of variance from 3 independent examinations. 12C, SN12C; 12L1, SN12L1; CDH1, E-cadherin; nCLDN4, nuclear CLDN4; C, untreated control; PKC-A, PKCε activator; PKC-I, PKCε inhibitor peptide.

As YAP activation has been reported to promote EMT [14,15], we examined the EMT phenotype in SN12C and SN12L1 cells with and without PKCε activator and PKCε inhibitor treatments, respectively (Figure 4A). In SN12C cells, the expression of E-cadherin was maintained and that of snail, vimentin, and CD44 was at low levels, whereas PKCε activator-treated SN12C cells showed decreased expression of E-cadherin and increased expression of snail, vimentin, and CD44. In contrast, in SN12L1 cells, E-cadherin expression was at low levels, while snail, vimentin, and CD44 expression was at high levels, whereas SN12L1 cells treated with PKCε inhibitor showed enhanced E-cadherin expression and reduced expression of snail, vimentin, and CD44. In an in vitro invasion assay, under non-treated conditions, the invasion ability of SN12L1 cells was approximately 3 times higher than that of SN12C cells (Figure 4B). The number of invading cells increased 1.9-fold when SN12C cells were treated with the PKCε activator, whereas the number decreased to approximately 1/2 when SN12L1 cells were treated with the PKCε inhibitor. We next examined the stemness of both cell lines by sphere-forming assay (Figure 4C). The number of spheres increased by 1.4-fold when SN12C cells were treated with the PKCε activator, while it decreased to approximately 1/2 when SN12L1 cells were treated with the PKCε inhibitor. Finally, we examined the metastability of both cell lines using a nude mice lung metastasis model (Figure 4D). The fluorescence intensity of metastasized cells increased by 1.8-fold when SN12C cells were treated with the PKCε activator, while it decreased to approximately 1/2 when SN12L1 cells were treated with the PKCε inhibitor.

## 3. Discussion

Previous reports have suggested that CLDN4 is expressed at low levels in RCC and in the bladder, colon, stomach, pancreatic, breast, and oral cancers [13,14,15,20,21,22,23]. In CCCs, which account for most of the cases examined in this study, VHL inactivation is widely observed [24]. VHL gene mutation reduces occludin and CLDN expression [25]. Furthermore, HIF1 activation caused by VHL gene inactivation suppresses E-cadherin expression [25,26].

In RCC, expression of CLDNs 1, 2, 3, 4, 5, 7, and 16 has been reported [27,28], and CLDN2 was observed to show high expression [27]. CLDN1 and 16 are downregulated with cancer progression [28]. CLDN1 and 2 expression correlates with tumor grade [27], and CLDN1, 3, and 4 expression is considered a poor prognostic factor [29,30]. In contrast, in our study, CLDN4 expression did not correlate with clinicopathologic factors of RCC.

In the present study, CLDN4 expression was observed in the nucleus in some RCC cases. In the previous reports, CLDN3, 4, 7, and 8 were found to be expressed in the cell membrane and cytoplasm, and CLDN1 was expressed in the cell membrane [30]. In our previous study, intranuclear CLDN4 was observed in approximately 30% of oral cancer cases. In these cases, *Clostridium perfringens* infection was detected to impair tight junction through CPE, which led to the intracytoplasmic translocation of CLDN4 in these tumors [14]. In the large intestine mucosa, tight junction impairment by CPE also causes CLDN4 translocation [21]. Furthermore, not only *Clostridium perfringens* but also *Shigella* infections impair the tight junction of the large intestine mucosa and alter CLDN2 and 4 localization, resulting in their accumulation in the cytoplasm [31]. From these findings, it is considered that release of CLDN4 from tight junctions induces alteration in the intracellular localization of CLDN4. The nuclear localization signal of CLDN4 is not clear. However, nuclear translocation of CLDN1, which lacks a nuclear localization signal, might translocate with binding with APC, ZO-1, or ZO-2 as shuttles [32].

Bacteria such as *Clostridium perfringens* that impair tight junctions were not found in the RCC cases that we examined (data not shown). Therefore, we investigated the phosphorylation of CLDN, which is known to impair tight junctions [33]. Our data showed that in SN12L1 cells showing CLDN4 nuclear localization, phosphorylation of serine and tyrosine residues was observed in cytoplasmic CLDN4, whereas only serine phosphorylation was observed in nuclear CLDN4. This suggests that tyrosine phosphorylation might be associated with cytoplasmic translocation and serine phosphorylation might be related to nuclear translocation. 

EphA2 is an enzyme known to phosphorylate the tyrosine residue of CLDN4 [18]. EphA2 binds to CLDN4 of tight junction, phosphorylates Tyr208, which is in an intracellular domain near the N-terminus of CLDN4, and reduces the binding of CLDN4 to ZO-1; this leads to the translocation of CLDN4 from the tight junction to the cytoplasm [18]. Our data showed that SN12L1 cells highly express EphA2 and its ligand, Ephrin A1. Inhibition of EphA2 reduced cytoplasmic CLDN4 levels. In contrast, in SN12C cells that show low expression of EphA2 and Ephrin A1, treatment with Ephrin A1 led to increased CLDN4 in the cytoplasm. EphA2 promotes RCC invasion and survival and is correlated with RCC grade, tumor size, and poor prognosis [34,35,36]. EphA2 also induces EMT in cancer cells [37].

PKCε is an enzyme that causes phosphorylation of serine/threonine residues of CLDN [33]. PKCε phosphorylates Thr189 and Ser194 in ovarian cancer, resulting in a decrease in tight junction binding [19]. There are no reports of other PKC isozymes using CLDN4 as a substrate. However, classical PKCs phosphorylate CLDN1 to induce nuclear localization in melanoma cells [38]. Our data showed that PKCε expression was high in SN12L1 cells, and inhibition of PKCε reduced nuclear CLDN4 expression levels. In contrast, SN12C cells with low PKCε expression also showed increased nuclear CLDN4 upon activation of PKCε. In these experiments, because cytoplasmic CLDN4 expression was also altered, PKCε-mediated serine phosphorylation might be associated with cytoplasmic translocation of CLDN4. 

PKCε is overexpressed in RCC, especially in CCC, and correlates with Fuhrman grade and tumor size [39]. PKCε is one of the novel PKCs and is known as a transforming oncogene and a tumor biomarker. PKCε activates phosphoinositide 3-kinase/Akt, extracellular-signal-regulated kinase signaling, and integrin β1 [40,41], which is associated with increased RCC stemness [40]. PKCε is activated by stress such as ultraviolet and radiation in addition to phorbol ester (12-O-tetradecanoylphorbol 13-acetate) [42,43]. Furthermore, PKCε and other novel PKCs are activated by 14-3-3ζ [33,44], which is highly expressed in RCC and is associated with metastasis and poor prognosis [45].

From these findings, CLDN4 phosphorylation by EphA2 and PKCε might cause tight junction impairment and release CLDN4 from tight junction and might enhance binding with YAP and ZO-1 to form nuclear translocating complex [15]. 

We have previously shown that the nuclear translocation of YAP bound to CLDN4 occurs with the nuclear translocation of CLDN4, resulting in the activation of YAP and EMT [14]. In this study, nuclear transfer of YAP bound to CLDN4 was observed in SN12L1 cells, and nuclear YAP was decreased by PKCε inhibition. In contrast, in SN12C cells, nuclear translocation of CLDN4 and YAP was not observed, whereas PKCε activation induced their nuclear translocation and EMT.

With the nuclear translocation of CLDN4 and YAP, the induction of the EMT phenotype was observed, and the invasive and metastatic abilities of the cells were also increased. In our previous studies, induction of the EMT phenotype by YAP activation was found in oral and colorectal cancers [14,15,16]. In addition, increased CD44 expression and enhanced sphere formation were observed, which suggest increased stemness in cancer cells. Furthermore, when a nude mouse lung metastasis model was examined, SN12L1 cell metastasis was suppressed by PKCε inhibition and, conversely, SN12C cell metastasis was promoted by PKCε activation. The SN12L1 cell line is a highly metastatic strain established from the SN12C cell line [46,47]. The EMT phenotype induced by the EphA2-PKCε-CLDN4-YAP axis is considered one of the mechanisms underlying the metastatic ability of SN12L1 cells. Thus, it was considered that the YAP activation associated with CLDN4 nuclear translocation plays an important role in the acquisition of a malignant phenotype in RCC. These findings are expected to provide new molecular targets for RCC treatment.

We found that CLDN4 phosphorylation by EphA2/Ephrin A1 and PKCε induced nuclear translocation of YAP with CLDN4 to provided EMT in RCC cells. Sarcomatoid RCC is not included in the 202 cases examined this time. Examining the nuclear translocation of CLDN4 in tumors exhibiting EMT phenotype such as sarcomatoid RCC is an interesting issue. It should be examined in the future. The frequency of nuclear translocation of CLDN4 is low, probably because it uses a method with low detection sensitivity by immunostaining. It is necessary to study using a highly sensitive method such as ELISA. The release of CLDN4 by impaired tight junction might be one of the mechanisms of malignant properties of RCC, which is expected to be a novel therapeutic target for RCC treatment.

## 4. Materials and Methods

### 4.1. Surgical Specimens

We reviewed the pathological diagnosis and clinical data of 202 patients with surgically resected RCC, reviewed at the Department of Molecular Pathology, Nara Medical University, during 2006–2015. As written informed consent was not obtained, any identifying information was removed from the samples before analysis to ensure strict privacy protection (unlinkable anonymization). All procedures were performed in accordance with the Ethical Guidelines for Human Genome/Gene Research enacted by the Japanese Government and were approved by the Ethics Committee of Nara Medical University (Approval Number 937, 2020/4/1).

### 4.2. Cell Lines

SN12C and SN12L1 human RCC cell lines were kindly provided by Professor Isaiah J Fidler (MD Anderson Cancer Center, TX, USA) [47]. Cells were cultured in Dulbecco’s modified Eagle’s medium supplemented with 10% fetal bovine serum at 37 °C in 5% CO_2_.

An in vitro invasion assay was performed using a type IV collagen-coated insert. The number of cells invading into the collagen membrane was measured after 48 h.

### 4.3. Sphere Assay

SN12C or SN12L1 cells (1000 cells per well) were seeded on uncoated bacteriological 35 mm-dish (Coning Inc., Coning, NY, USA) with 3D Tumorsphere Medium XF (Sigma-Aldrich Inc., St. Louis, MO, USA). After 5 days, the sphere number was counted.

### 4.4. Antibody and Reagents

Anti-human CLDN4 extracellular domain antibody, 4D3, was developed by immunizing rats with a plasmid vector encoding human CLDN4 [20]. Anti-EphA2 antibody (clone 1A9C3, 1 µg/mL for blocking concentration, Proteintech Group Inc., Rosemont, IL, USA), PKCε inhibitor peptide (10 μM for working concentration, sc-3095, Santa-Cruz Biotechnology, Santa-Cruz, CA, USA), L-alpha-phosphatidylinositol-3,4,5-trisphosphate sodium salt (PKCδ/ε/η activator, 5 µM for working concentration, ab145221, Abcam, Cambridge, MA, USA), and recombinant human ephrin A1 protein (10 µg/mL for working concentration, ab181919, Abcam) were purchased.

### 4.5. Immunohistochemistry

Consecutive 4-mm sections were immunohistochemically stained using anti-CLDN4 antibody (0.2 µg/mL, clone 4D3), which was established in our laboratory [20], and a previously described immunoperoxidase technique [48] was performed. Secondary antibodies for peroxidase-conjugated mouse IgG and alkaline phosphatase-conjugated rabbit IgG (Medical and Biological Laboratories, Nagoya, Japan) were used at a concentration of 0.2 µg/mL. Tissue sections were color-developed with diamine benzidine hydrochloride (DAKO, Glastrup, Denmark). Slides were counterstained with Meyer’s hematoxylin (Sigma). We counted immunopositive cells at the cytoplasmic membrane. Staining strength was scored from 0 to 3 (a score of 1 was used to describe the expression level in normal renal tubule epithelium). The staining index was calculated as the staining strength score multiplied by the staining area (%). As negative control, non-immunized rat IgG (Santa-Cruz) was used as the primary antibody.

### 4.6. Protein Extraction

For preparing whole-cell lysate, SN12C and SN12L1 cells were washed twice with cold phosphate-buffered saline (PBS), harvested, and lysed with 0.1% sodium dodecyl sulfate (SDS)-added radioimmunoprecipitation assay buffer (Thermo Fisher Scientific, Tokyo, Japan) [49]. Cell fractions were extracted using a Cell Fractionation Kit (Abcam), according to the manufacturer’s instructions [50]. Protein assay was performed using a Protein Assay Rapid Kit (Wako Pure Chemical Corporation, Osaka, Japan).

### 4.7. Immunoblot Analysis

Lysates (20 μg) were subjected to immunoblot analysis using SDS–polyacrylamide gel electrophoresis (12.5%), followed by electrotransfer onto nitrocellulose filters. The filters were incubated with primary antibodies, followed by peroxidase-conjugated IgG antibodies (Medical and Biological Laboratories). Anti-tubulin antibody was used to assess the protein levels loaded per lane (Oncogene Research Products, Cambridge, MA, USA). The immune complex was visualized using an enhanced chemiluminescence Western blot detection system (Amersham, Aylesbury, UK). Antibodies for E-cadherin (DAKO), CLDN4 (clone 4D3) [20], EphA2, Ephrin A1, vimentin (Proteintech), snail (Biorbyt, St Louis, MO, USA), YAP1, CD44 (Abcam, Cambridge, UK), and PKCε (Enzo Lifesciences, Inc., Farmingdale, NY, USA) were used as primary antibodies. Tubulin (Zymed Laboratories Inc., South San Francisco, CA, USA) and lamin (Proteintech) were used as the loading control.

### 4.8. Immunoprecipitation

Immunoprecipitation was performed according to the method described previously [51]. Briefly, whole-cell lysates were pre-cleaned in lysis buffer with protein A/G agarose (Santa-Cruz) for 1 h at 4 °C and subsequently centrifuged. The supernatants were incubated with antibody against CLDN4 (4D3) and protein A/G agarose for 3 h at 4 °C. Precipitates were collected by centrifugation, washed five times with lysis buffer, solubilized with sample buffer (40 µL; Sigma), and subjected to immunoblot analysis with antibodies against YAP1 (Abcam) or phosphoserine (Abcam, ab9332), or phosphotyrosine (Abcam, ab179530). Loading protein volume was confirmed by slot blot analysis of 10 µL of the samples by Coomassie blue staining (Bio-Rad, Hercules, CA, USA).

### 4.9. Enzyme-Linked Immunosorbent Assay (ELISA) and Colorimetric Assay

An ELISA kit was used to measure the concentration of human CLDN4 (Cusabio Biotech Co., Ltd., Houston, TX, USA). The assay was performed according to the manufacturers’ instructions, and whole-cell lysates were used for the measurements.

### 4.10. Animals

BALB/c nude mice (4 weeks old, male) were purchased from SLC Japan (Shizuoka, Japan). The mice were maintained according to the institutional guidelines approved by the Committee for Animal Experimentation of Nara Medical University, in accordance with the current regulations and standards of the Ministry of Health, Labor, and Welfare (Approval number 12047, 2017/7/20).

### 4.11. Lung Metastasis Model

SN12L1 and SN12C cells were pretreated with or without PKC-I (10 μM) and PKC-A (5 µM), respectively, for 24 h. Suspensions (1 × 10^6^ cells/50 μL of PBS) of SN12L1 and SN12C cells labeled with VivoTrack 680 (PerkinElmer Inc., Waltham, MA, USA) were injected into the caudal vein. The mice were euthanized, and the lungs were observed using the Clairvivo OPT in vivo imager (Shimazu, Kyoto, Japan) two weeks after inoculation.

### 4.12. Statistical Analysis

Statistical significance was calculated using two-tailed Fisher’s exact test, an ordinary analysis of variance, and InStat software version 3.0 (GraphPad, Los Angeles, CA, USA). A two-sided *p* value of <0.05 was considered to indicate statistical significance.

## Figures and Tables

**Table 1:** Expression of membranous CLDN4 in 202 cases of renal cell carcinomas.

Parameter ^1^		*n*	Expression of CLDN4 ^2^	*p* ^3^
**Age**	−60 y	52	53 ± 8	NS
	61 y -	150	56 ± 6	
**Sex**	Male	111	56 ± 9	NS
	Female	91	54 ± 7	
**Histology**	Clear cell	200	55 ± 9	NS
	Papillary	2	52 ± 10	
**Grade**	1	161	52 ± 7	NS
	2	34	59 ± 6	
	3	7	41 ± 7	
**pT**	1	145	50 ± 6	NS
	2	46	64 ± 7	
	3	11	49 ± 7	
**pN**	0	192	57 ± 8	NS
	1	10	55 ± 8	
**Stage**	I	146	49 ± 6	NS
	II	44	68 ± 8	
	III-IV	12	54 ± 7	

^1^ Clinicopathological classification is according to the Union for International Cancer Control-tumor/node/metastasis UICC-TNM Classification [17]. Clear cell, clear cell renal cell carcinoma; Papillary, papillary renal cell carcinoma; pT1, Tumor ≤7 cm in greatest dimension, limited to the kidney; pT2, Tumor >7 cm in greatest dimension, limited to the kidney; pT3, Tumor extends into major veins or perinephric tissues but not into the ipsilateral adrenal gland and not beyond the Gerota fascia; pN0, No regional lymph node metastasis; pN1, Metastasis in regional lymph node(s); stage I, pT1/pN0/M0; stage II, pT2/pN0/M0; stage III, pT2/pN1/M0 or pT3/pNany/M0; stage IV, pT4/pNany/M0; M, distant metastasis. ^2^ Claudin-4 expression index was calculated as the staining strength score multiplied by the staining area. ^3^ *P* value was calculated using Student’s *t*-test; NS, no significant correlation.

**Table 2:** Nuclear CLDN4 expression in renal cell carcinomas.

CLDN4	*n*	pT3 ^1)^		*p* ^2)^
**Nuclear**	5	5	(100%)	
**Non-nuclear**	197	6	(3%)	<0.0001

1) Clinicopathological classification is according to the UICC-TNM Classification [17]. pT3, Tumor extends into major veins or perinephric tissues but not into the ipsilateral adrenal gland and not beyond the Gerota fascia. 2) *p* value was calculated using Student’s *t*-test.

## Abbreviation

RCC	renal cell carcinoma;
CCC	clear cell renal cell carcinoma
HIF	hypoxia-inducible factor
VHL	von Hippel-Lindau
EMT	epithelial-mesenchymal transition
CLDN	claudin
CPE	*Clostridium perfringens* enterotoxin
YAP	Yes-associated protein
PKC	protein kinase C

## References

[1] Tamaki T., Dong Y., Ohno Y., Sobue T., Nishimoto H., Shibata A. (2014). The burden of rare cancer in Japan: Application of the RARECARE definition. Cancer Epidemiol..

[2] Motzer R.J., Bander N.H., Nanus D.M. (1996). Renal-cell carcinoma. N. Engl. J. Med..

[3] Buchner A., Riesenberg R., Kotter I., Hofstetter A., Stief C., Oberneder R. (2006). Frequency and prognostic relevance of disseminated tumor cells in bone marrow of patients with metastatic renal cell carcinoma. Cancer.

[4] Hsieh J.J., Le V.H., Oyama T., Ricketts C.J., Ho T.H., Cheng E.H. (2018). Chromosome 3p Loss-Orchestrated VHL, HIF, and Epigenetic Deregulation in Clear Cell Renal Cell Carcinoma. J. Clin. Oncol..

[5] Braga E.A., Fridman M.V., Loginov V.I., Dmitriev A.A., Morozov S.G. (2019). Molecular Mechanisms in Clear Cell Renal Cell Carcinoma: Role of miRNAs and Hypermethylated miRNA Genes in Crucial Oncogenic Pathways and Processes. Front. Genet..

[6] Rankin E.B., Fuh K.C., Castellini L., Viswanathan K., Finger E.C., Diep A.N., LaGory E.L., Kariolis M.S., Chan A., Lindgren D. (2014). Direct regulation of GAS6/AXL signaling by HIF promotes renal metastasis through SRC and MET. Proc. Natl. Acad. Sci. USA.

[7] Tsukita S., Furuse M., Itoh M. (2001). Multifunctional strands in tight junctions. Nat. Rev. Mol. Cell Biol..

[8] Turksen K., Troy T.C. (2011). Junctions gone bad: Claudins and loss of the barrier in cancer. Biochim. Biophys. Acta.

[9] Lin X., Shang X., Manorek G., Howell S.B. (2013). Regulation of the Epithelial-Mesenchymal Transition by Claudin-3 and Claudin-4. PLoS ONE.

[10] Fredriksson K., Van Itallie C.M., Aponte A., Gucek M., Tietgens A.J., Anderson J.M. (2015). Proteomic analysis of proteins surrounding occludin and claudin-4 reveals their proximity to signaling and trafficking networks. PLoS ONE.

[11] Li W., Xu C., Wang K., Ding Y., Ding L. (2019). Non-tight junction-related function of claudin-7 in interacting with integrinβ1 to suppress colorectal cancer cell proliferation and migration. Cancer Manag. Res..

[12] Lu Z., Kim D.H., Fan J., Lu Q., Verbanac K., Ding L., Renegar R., Chen Y.H. (2015). A non-tight junction function of claudin-7-Interaction with integrin signaling in suppressing lung cancer cell proliferation and detachment. Mol. Cancer.

[13] Nishiguchi Y., Fujiwara-Tani R., Sasaki T., Luo Y., Ohmori H., Kishi S., Mori S., Goto K., Yasui W., Sho M. (2019). Targeting claudin-4 enhances CDDP-chemosensitivity in gastric cancer. Oncotarget.

[14] Nakashima C., Yamamoto K., Kishi S., Sasaki T., Ohmori H., Fujiwara-Tani R., Mori S., Kawahara I., Nishiguchi Y., Mori T. (2020). Clostridium perfringens enterotoxin induces claudin-4 to activate YAP in oral squamous cell carcinomas. Oncotarget.

[15] Fujiwara-Tani R., Fujii K., Mori S., Kishi S., Sasaki T., Ohmori H., Nakashima C., Kawahara I., Nishiguchi Y., Mori T. (2020). Role of Clostridium perfringens Enterotoxin on YAP Activation in Colonic Sessile Serrated Adenoma/ Polyps with Dysplasia. Int. J. Mol. Sci..

[16] Sasaki T., Mori S., Kishi S., Fujiwara-Tani R., Ohmori H., Nishiguchi Y., Hojo Y., Kawahara I., Nakashima C., Fujii K. (2020). Effect of Proton Pump Inhibitors on Colorectal Cancer. Int. J. Mol. Sci..

[17] Sobin L.H., Wittekind C. (2003). UICC TNM Classification of Malignant Tumours.

[18] Tanaka M., Kamata R., Sakai R. (2005). EphA2 phosphorylates the cytoplasmic tail of Claudin-4 and mediates paracellular permeability. J. Biol. Chem..

[19] D’Souza T., Indig F.E., Morin P.J. (2007). Phosphorylation of claudin-4 by PKCepsilon regulates tight junction barrier function in ovarian cancer cells. Exp. Cell Res..

[20] Kuwada M., Chihara Y., Luo Y., Li X., Nishiguchi Y., Fujiwara R., Sasaki T., Fujii K., Ohmori H., Fujimoto K. (2015). Pro-chemotherapeutic effects of antibody against extracellular domain of claudin-4 in bladder cancer. Cancer Lett..

[21] Fujiwara-Tani R., Sasaki T., Luo Y., Goto K., Kawahara I., Nishiguchi Y., Kishi S., Mori S., Ohmori H., Kondoh M. (2018). Anti-claudin-4 extracellular domain antibody enhances the antitumoral effects of chemotherapeutic and antibody drugs in colorectal cancer. Oncotarget.

[22] Sasaki T., Fujiwara-Tani R., Kishi S., Mori S., Luo Y., Ohmori H., Goto K., Nishiguchi Y., Mori T., Sho M. (2019). Targeting claudin-4 enhances chemosensitivity of pancreatic ductal carcinomas. Cancer Med..

[23] Luo Y., Kishi S., Sasaki T., Ohmori H., Fujiwara-Tani R., Mori S., Goto K., Nishiguchi Y., Mori T., Kawahara I. (2020). Targeting claudin-4 enhances chemosensitivity in breast cancer. Cancer Sci..

[24] Gossage L., Eisen T., Maher E.R. (2015). VHL, the story of a tumour suppressor gene. Nat. Rev. Cancer.

[25] Harten S.K., Shukla D., Barod R., Hergovich A., Balda M.S., Matter K., Esteban M.A., Maxwell P.H. (2009). Regulation of renal epithelial tight junctions by the von Hippel-Lindau tumor suppressor gene involves occludin and claudin 1 and is independent of E-cadherin. Mol. Biol. Cell.

[26] Ajduković J. (2016). HIF-1--a big chapter in the cancer tale. Exp. Oncol..

[27] Virman J., Soini Y., Kujala P., Luukkaala T., Salminen T., Sunela K., Kellokumpu-Lehtinen P.L. (2014). Claudins as prognostic factors for renal cell cancer. Anticancer Res..

[28] Men W., Martin T.A., Ruge F., Zhang N., Du P., Yang Y., Jiang W.G. (2015). Expression of claudins in human clear cell renal cell carcinoma. Cancer Genom. Proteom..

[29] Fritzsche F.R., Oelrich B., Johannsen M., Kristiansen I., Moch H., Jung K., Kristiansen G. (2008). Claudin-1 protein expression is a prognostic marker of patient survival in renal cell carcinomas. Clin. Cancer Res..

[30] Lechpammer M., Resnick M.B., Sabo E., Yakirevich E., Greaves W.O., Sciandra K.T., Tavares R., Noble L.C., DeLellis R.A., Wang L.J. (2008). The diagnostic and prognostic utility of claudin expression in renal cell neoplasms. Mod. Pathol..

[31] Sarkar P., Saha T., Sheikh I.A., Chakraborty S., Aoun J., Chakrabarti M.K., Rajendran V.M., Ameen N.A., Dutta S., Hoque K.M. (2019). Zinc ameliorates intestinal barrier dysfunctions in shigellosis by reinstating claudin-2 and -4 on the membranes. Am. J. Physiol. Gastrointest Liver Physiol..

[32] Dhawan P., Singh A.B., Deane N.G., No Y., Shiou S.R., Schmidt C., Neff J., Washington M.K., Beauchamp R.D. (2005). Claudin-1 regulates cellular transformation and metastatic behavior in colon cancer. J. Clin. Investig..

[33] McCole D.F. (2013). Phosphatase regulation of intercellular junctions. Tissue Barriers.

[34] Cho M.C., Cho S.Y., Yoon C.Y., Lee S.B., Kwak C., Kim H.H., Jeong H. (2015). EphA2 Is a Potential Player of Malignant Cellular Behavior in Non-Metastatic Renal Cell Carcinoma Cells but Not in Metastatic Renal Cell Carcinoma Cells. PLoS ONE.

[35] Xu J., Zhang J., Cui L., Zhang H., Zhang S., Bai Y. (2014). High EphA2 protein expression in renal cell carcinoma is associated with a poor disease outcome. Oncol. Lett..

[36] Herrem C.J., Tatsumi T., Olson K.S., Shirai K., Finke J.H., Bukowski R.M., Zhou M., Richmond A.L., Derweesh I., Kinch M.S. (2005). Expression of EphA2 is prognostic of disease-free interval and overall survival in surgically treated patients with renal cell carcinoma. Clin. Cancer Res..

[37] Fattet L., Jung H.Y., Matsumoto M.W., Aubol B.E., Kumar A., Adams J.A., Chen A.C., Sah R.L., Engler A.J., Pasquale E.B. (2020). Matrix Rigidity Controls Epithelial-Mesenchymal Plasticity and Tumor Metastasis via a Mechanoresponsive EPHA2/LYN Complex. Dev. Cell.

[38] French A.D., Fiori J.L., Camilli T.C., Leotlela P.D., O’Connell M.P., Frank B.P., Subaran S., Indig F.E., Taub D.D., Weeraratna A.T. (2009). PKC and PKA phosphorylation affect the subcellular localization of claudin-1 in melanoma cells. Int. J. Med. Sci..

[39] Huang B., Cao K., Li X., Guo S., Mao X., Wang Z., Zhuang J., Pan J., Mo C., Chen J. (2011). The expression and role of protein kinase C (PKC) epsilon in clear cell renal cell carcinoma. J. Exp. Clin. Cancer Res..

[40] Huang B., Fu S.J., Fan W.Z., Wang Z.H., Chen Z.B., Guo S.J., Chen J.X., Qiu S.P. (2016). PKCε inhibits isolation and stemness of side population cells via the suppression of ABCB1 transporter and PI3K/Akt, MAPK/ERK signaling in renal cell carcinoma cell line 769P. Cancer Lett..

[41] Brenner W., Benzing F., Gudejko-Thiel J., Fischer R., Färber G., Hengstler J.G., Seliger B., Thüroff J.W. (2004). Regulation of beta1 integrin expression by PKCepsilon in renal cancer cells. Int. J. Oncol..

[42] Aziz M.H., Manoharan H.T., Sand J.M., Verma A.K. (2007). Protein kinase Cepsilon interacts with Stat3 and regulates its activation that is essential for the development of skin cancer. Mol. Carcinog..

[43] Kim C.Y., Giaccia A.J., Strulovici B., Brown J.M. (1992). Differential expression of protein kinase C epsilon protein in lung cancer cell lines by ionising radiation. Br. J. Cancer.

[44] Acs P., Szallasi Z., Kazanietz M.G., Blumberg P.M. (1995). Differential activation of PKC isozymes by 14–3-3 zeta protein. Biochem. Biophys. Res. Commun..

[45] Masui O., White N.M., DeSouza L.V., Krakovska O., Matta A., Metias S., Khalil B., Romaschin A.D., Honey R.J., Stewart R. (2013). Quantitative proteomic analysis in metastatic renal cell carcinoma reveals a unique set of proteins with potential prognostic significance. Mol. Cell Proteom..

[46] Anzai H., Kitadai Y., Bucana C.D., Sanchez R., Omoto R., Fidler I.J. (1996). Intratumoral heterogeneity and inverse correlation between expression of E-cadherin and collagenase type IV in human gastric carcinomas. Differentiation.

[47] Naito S., von Eschenbach A.C., Fidler I.J. (1987). Different growth pattern and biologic behavior of human renal cell carcinoma implanted into different organs of nude mice. J. Natl. Cancer Inst..

[48] Kuniyasu H., Oue N., Wakikawa A., Shigeishi H., Matsutani N., Kuraoka K., Ito R., Yokozaki H., Yasui W. (2002). Expression of receptors for advanced glycation end-products (RAGE) is closely associated with the invasive and metastatic activity of gastric cancer. J. Pathol..

[49] Kuniyasu H., Yano S., Sasaki T., Sasahira T., Sone S., Ohmori H. (2005). Colon cancer cell-derived high mobility group 1/amphoterin induces growth inhibition and apoptosis in macrophages. Am. J. Pathol..

[50] Matsushima-Otsuka S., Fujiwara-Tani R., Sasaki T., Ohmori H., Nakashima C., Kishi S., Nishiguchi Y., Fujii K., Luo Y., Kuniyasu H. (2018). Significance of intranuclear angiotensin-II type 2 receptor in oral squamous cell carcinoma. Oncotarget.

[51] Kuniyasu H., Yasui W., Pettaway C.A., Yano S., Oue N., Tahara E., Fidler I.J. (2001). Interferon-alpha prevents selection of doxorubicin-resistant undifferentiated- androgen-insensitive metastatic human prostate cancer cells. Prostate.

